# Validation of the rating scales for negative symptoms: new strategies

**DOI:** 10.1192/j.eurpsy.2024.75

**Published:** 2024-08-27

**Authors:** S. Leucht, S. Wehr

**Affiliations:** Psychiatry and Pwsychotherapy, Technical University of Munich, School of Medicine and Health, Munich, Germany

## Abstract

**Abstract:**

Negative symptoms of schizophrenia are linked with poor functioning and quality of life. Therefore, appropriate measurement tools to assess negative symptoms are needed. The NIMH-MATRICS Consensus defined five domains for negative symptoms. We used the COSMIN guidelines for systematic reviews to evaluate the quality of psychometric data of negative symptom scales as Clinician-Rated Outcome Measure (ClinROM). COSMIN assesses risk of bias, so called updated criteria of measurement properties, a modified GRADE approach and a final judgement on the rating scale. In the lecture the process will be described using the Brief Negative Symptom Scale and the Clinical Assessment Interview for Negative Symptoms (CAINS) as examples.

**Disclosure of Interest:**

None Declared

